# Self-perceived postural balance correlates with postural balance and anxiety during the first year after stroke: a part of the randomized controlled GOTVED study

**DOI:** 10.1186/s12883-020-01982-z

**Published:** 2020-11-09

**Authors:** Lena Rafsten, Anna Danielsson, Katharina S. Sunnerhagen

**Affiliations:** 1 Department of Clinical Neuroscience, Institute of Neuroscience and Physiology, Sahlgrenska Academy, University of Gothenburg, Per Dubbsgatan 14, fl. 3, 413 45 Gothenburg, Sweden; 2 Department of Occupational Therapy and Physiotherapy, Sahlgrenska University Hospital, Gothenburg, Sweden; 3 Centre for Person-Centred Care (GPCC), University of Gothenburg, Gothenburg, Sweden; 4 Department of Health and Rehabilitation, Institute of Neuroscience and Physiology, Sahlgrenska Academy, University of Gothenburg, Gothenburg, Sweden

**Keywords:** Stroke, Postural-balance, Anxiety, Self-confidence, Outcome measure

## Abstract

**Background:**

Postural balance is an important rehabilitation outcome, and screening stroke patients for confidence in postural balance during rehabilitation and before hospital discharge is recommended. Early supported discharge could improve postural balance self-confidence.

This study aimed to investigate associations between patient self-confidence in postural balance and observer-assessed postural balance and anxiety during the first year after stroke. Whether very early supported discharge (VESD) affects self-confidence in postural balance compared with standard discharge was also evaluated.

**Methods:**

A longitudinal trial for with data extracted from a randomized controlled study of 140 adults with confirmed stroke was conducted. The experimental group received VESD. The control group was discharged according to the standard routine. Postural balance was assessed with Berg Balance Scale (BBS), Timed Up and Go (TUG) test, and Falls Efficacy Scale. Anxiety was assessed with the Hospital Anxiety and Depression Scale. Spearman’s rank correlation coefficient (rho) was used to test associations between independent variables. The Wilcoxon signed-rank test was used to examine differences over time. A single test, according to Eid, Gollwitzer, and Schmidt, was used to test temporal differences in correlation.

**Results:**

The correlation between self-confidence in postural balance and observer-assessed postural balance was 0.62–0.78 in the first year after stroke. The correlation between self-confidence and anxiety was 0.22–0.41 in the first year after stroke. Correlations did not differ by group affiliation at any time point when the postural balance was assessed with BBS. The intervention group had a significantly higher correlation (*r* = − 0.709) than the control group (*r* = − 0.416) when postural balance was assessed with the TUG test 1 month after discharge. There were no significant differences in correlations between confidence in postural balance and anxiety between the two groups at any time point.

**Conclusions:**

Patients with mild stroke can accurately assess their confidence in performing daily activities without falling. VESD does not substantially affect the correlation between self-confidence in postural balance and observer assessed postural balance and is safe to use as an alternative to standard discharge. Assessment of self-confidence can provide important information for rehabilitation planning and supporting the physical activity of patients after discharge.

**Trial registration:**

Clinical Trials.gov: NCT01622205. Registered 19 June 2012 (retrospectively registered).

## Background

Impaired muscle strength, sensation, postural balance, emotion and cognition is common symptoms after stroke, which may restrict the patient’s ability to perform activities of daily living (ADL). This makes stroke one of the leading causes of permanent disability in adults worldwide [1].

Postural balance is essential for body control during various body movements in diverse activities [2]. Patient suffering from impaired postural balance after a stroke have an increased risk of falling [3, 4]. Rates of falling are nearly double among people with stroke than among age- and sex-matched counterparts, and some people with stroke have even reported that up to 70% fall in the first year after stroke [5, 6]. The fall rate seems to be higher upon discharge from the hospital.

Impairments in postural balance and mobility are associated with decreased confidence in postural balance [7]. Confidence regarding self-efficacy in postural balance may be an important factor contributing to stroke recovery, as it has been found to mediate outcomes such as engagement in activities of daily living [8]. The correlation between falls and fear of falling has been well demonstrated [9–13]. However, the causal relationship between these two factors is unclear. Falls can cause fear of falling, fear of falling can cause falls, and the two outcomes may be related to other shared risk factors and not causally related. Studies showed that patients with poor self-efficacy were twice as likely to fall in the subsequent 12 months than those with better balance self-efficacy scores [14].

Less attention has been given to the effects of stroke recovery on psychological factors that, for example, are due to postural balance. After stroke onset, anxiety is a common symptom in the acute phase, months and years later. A systematic review from 2018 showed that 29% of stroke survivors experienced anxiety at any time during the first year after stroke [15]. Anxiety is associated with a decreased quality of life [16]. Anxiety receives significantly less attention than other psychological problems after stroke [17], and a large-scale survey reported dissatisfaction with the provision of psychological services after stroke [18].

Postural balance confidence is an independent predictor of perceived physical function, perceived mobility, and perceived recovery 1 year after inpatient stroke rehabilitation [19]. Postural balance is remediable and a relevant rehabilitation outcome; therefore, stroke patients should be screened for reduced confidence in postural balance before discharge from the hospital. To enable prompt rehabilitation to avoid unnecessary falling, it is recommended that postural balance confidence be assessed during continued stroke rehabilitation [7].

Researchers have explored the discharge process following stroke to determine if developing new services would make the process more effective [20]. Early supported discharge (ESD) is a service that is suggested to be more effective after discharge following stroke. There are different ways to improve the patient’s self-confidence in, for example, postural balance-efficacy [21–23]. ESD might be a way to improve balance self-confidence. Previous studies have investigated how depression and walking ability affect self-confidence in postural balance, but no study has addressed how anxiety and ESD after stroke might affect self-confidence in postural balance.

The research questions for this study were as follows:

1. What are the associations between patients’ self-confidence in postural balance and observer-assessed postural balance and anxiety during the first year after stroke?
2. Does very early supported discharge (VESD) change self-confidence in postural balance after stroke when compared with the usual discharge routine?

## Method

### Study design

**Fig. 1:**
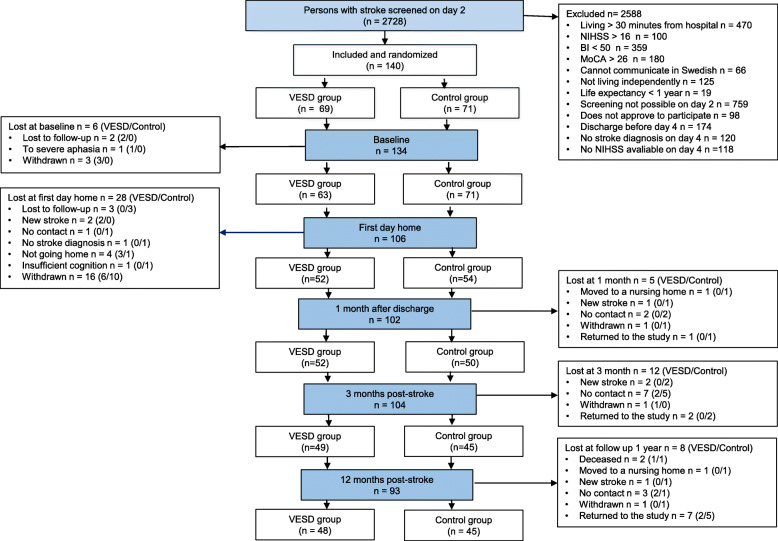
Design and flow of participants through the study

The study is a longitudinal trial for which the data were extracted from a randomized controlled study, Gothenburg Very Early Supported Discharge (GOTVED) [24] clinicaltrials.gov: NCT01622205. Over a period of 5 years, from September 2011 to April 2016, 140 adult patients from a stroke unit at the Sahlgrenska University Hospital were consecutively included in the study (Fig. 1). The CONSORT checklist was followed [25]. The allocation was prepared by an external person by supplying group allocation in an envelope and then sealing, mixing and numbering the slips. The inclusion was made by a research coordinator who informed the blinded assessor and the stroke team nurse about the new patient. The assessor worked at another ward at the hospital, minimizing the risk of the assessor learning the allocation by chance. The participants and the rehabilitation team were not blinded. The intervention took place in the patient’s home for 4 weeks after discharge. The assessments were made at the stroke unit 5 days after admission (baseline), 1 day after discharge in the patient’s home (as with the following assessments), 1 month after discharge, and 3 and 12 months post-stroke.

### Study population

As stated in the published study [26] an experienced physiotherapist who was blinded assessed participants. The inclusion criteria were: ischaemic or haemorrhagic stroke confirmed according to World Health Organization (WHO) criteria [27]; age 18 years or older; residence within 30 min by car of the stroke unit; a National Institute of Health Stroke Scale (NIHSS) [28, 29] score of 0–16 points, which corresponds to mild-to-moderate stroke [30]; a Barthel Index (BI) [31] score of 50 points or more on day 2 [32]; and a Montreal Cognitive Assessment [33] index of 26 points or less if BI = 100. Patients with a life expectancy < 1 year (e.g., with severe malignancy) or who could neither speak nor communicate in Swedish prior to stroke were excluded. The stroke subtypes were confirmed by imaging, and treatment with thrombolysis or thrombectomy was recorded. Of the 140 patients included in the study, 69 were randomized to the VESD group, and 71 were randomized to the control group. There was a difference in group size because one patient was unfortunately randomly allocated in error.

### Outcome measures

Postural balance was objectively assessed using the Berg Balance Scale (BBS) [34–36] and the Timed Up and Go (TUG) test [37]. The BBS has been shown to be an appropriate screening tool to predict fall risks with moderate accuracy [38]. To identify patients with impaired postural balance according to the BBS, we chose a BBS score < 45 [35, 39] as the cut-off. The TUG test is commonly used to examine functional mobility, reflecting postural balance and gait manoeuvres used in daily life [37]. The TUG test was performed twice, and the score from the second test was used. The use of walking aids was recorded. In the present study, we adopted a TUG cut-off score of > 15 s [39, 40] to identify impaired postural balance. Some patients were not able to perform the TUG test (4 patients in the VESD group and 5 patients in the control group) due to poor postural balance, and were then registered as having impaired postural balance.

Self-efficacy is defined as “an individual’s judgement of his or her ability to organize and execute given types of performances” [41]. In this study, we chose to use the term “patient confidence in postural balance”. Patient confidence in postural balance was explored using the Falls Efficacy Scale Swedish version (FES(S)) [42]. The FES is a questionnaire designed to measure self-perceived fear of falling during the performance of activities [43]. The FES(S) has shown high test-retest reliability and has also been found to be responsive to changes [44].

Anxiety was assessed by the Hospital Anxiety and Depression Scale-Anxiety subscale (HADS-A) [45]. The HADS is a 14-item self-assessment scale. Seven items of the HADS assess anxiety (score 0–21) and seven items assess depression (score 0–21). A higher score indicates higher distress for each subscale [45]. The questionnaires were administered in the presence of the researcher who could answer any questions if the patient did not understand the form.

### Intervention

The 69 patients allocated to the VESD group received continued rehabilitation in their homes from a rehabilitation team consisting of a physiotherapist, an occupational therapist, and a stroke nurse from the stroke care unit. To plan the rehabilitation, the patients were asked to formulate their goals at a goal-setting meeting prior to discharge, and after that, an individual rehabilitation programme was designed. Common goals were to be able to go out and buy food, take care of the laundry, travel by bus or tram, and manage the bills. The patients who received VESD received 2–4 visits per week by the physiotherapist and/or occupational therapist and if necessary 1–2 visits by the stroke nurse*,* with a maximum length of 4 weeks. In addition to training, the intervention also included tips on various activities to learn how to handle and adapt to different everyday activities and situations. If needed, the patients were referred to the outpatient rehabilitation team who would carry on the rehabilitation when discharged from the VESD.

### Control group

Seventy-one patients were randomized to the control group. They were discharged when they were medically stable and no longer in need of stroke unit care. In accordance with the stroke unit’s usual discharge routines, the patients had neither a goal-setting meeting nor a follow-up by the stroke team, but they could, if necessary, be referred to continued outpatient rehabilitation [24].

### Statistical analysis

Demographic data as well as stroke-related variables were expressed in percentages, mean ± SD, or median and interquartile range (IQR). The level of significance was set at *p* ≤ 0.05. For group differences in descriptive data, chi-squared test and Mann-Whitney U-test were used. Spearman’s rank correlation coefficient (rho) was used to test associations between the independent variables. Spearman’s rank correlation coefficient was used to test the correlation between the NIHSS total score and item scores (item numbers 3, 7, 8 and 11), the side of the lesion, the BBS score, the TUG score and the FES score. The strength of correlation were interpreted as small (*r < ±*0.29), medium (*r* = ± 0.30 to ±0.49) or large (*r* = ± 0.50 to 1.0) [46]. To test associations between the dichotomised and independent variables the Kendall’s rank correlation test was used. The responsiveness of the FES(S), BBS, and TUG test was examined by using the Wilcoxon signed-rank test to evaluate the change between each time point. Bonferroni correction of the *p*-value was used to correct testing between the time points [47]. The effect size for change scores was calculated by dividing the z value obtained from the Wilcoxon signed-rank test with the square root of the number of observations. Effect size values < 0.3 indicated small effects, values 0.30–0.49 indicated medium effects and ≤ 0.50 indicated large effects [47]. To test the changes in correlation over time between self-confidence in postural balance assessed with the FES(S), the observer assessed postural balance measured with the BBS and TUG test, and anxiety was measured with the HADS-A between the two groups using a single test, according to Eid, Gollwitzer, and Schmidt [48].

## Results

One hundred forty patients were consecutively enrolled in the study. The flow of the participants through the study is presented in Fig. 1. The mean age was 74 years (SD 11.8), and 62% of the patients were men (Table 1). There was no significant group difference in the descriptive data (Table 1). Of the participants, 36% had impaired postural balance according to the BBS, and 48% had impaired postural balance according to the TUG test at baseline. The effect sizes for the changes in the BBS and TUG scores varied from 0.02 to 0.49 (Table 2), and for all the effect sizes, a decreasing trend was noted over the late response period. At baseline, a negligible correlation was seen between the NIHSS total and items scores, and the side of the lesion, as well as between the side of the lesion and postural balance and anxiety. The correlations between the NIHSS item and total scores and the BBS, TUG and FES scores were also negligible at baseline. The correlation analysis by group affiliation showed a medium correlation between the lesion side and the TUG score (*r* = 0.342) in the control group. In the comparison of the correlation between the side of the lesion and the TUG score between the two groups, there was no statistically significant difference.

**Table 1:** Characteristics of the study population

Characteristics	All (***n*** = 140)	VESD (***n*** = 69)	Controls (***n*** = 71)	***p***
**Female/Male**, n	54/86	27/42	27/44	1.00
**Age**, years mean (SD)	74 (11.8)	75 (11)	73 (12)	0.23
**Stroke subtype**, n				
Ischemic stroke/ Intracerebral haemorrhage	129/10 (*n* = 139)	65/3 (*n* = 68)	64/7	0.32
**Localisation,** n (%)				
Right hemisphere	43 (30.7)	22 (31.9)	21 (29.6)	
Left hemisphere	35 (25.0)	16 (23.2)	19 (26.8)	
Unknown	41 (29.3)	20 (28.9)	21 (29.6)	
Bilateral	7 (5.0)	4 (5.8)	3 (4.2)	
Cerebellum	10 (7.1)	4 (5.8)	6 (8.5)	
Brain stem	4 (2.9)	3 (4.3)	1 (1.4)	
**Treatment**, n				
Thrombolysis/Thrombectomy	15/6	6/3	9/3	0.58/1.00
**NIHSS**^**a**^, median (IQR)	3 (1–5) (*n* = 137)	3 (1–5) (*n* = 68)	2 (1–5) (*n* = 69)	0.27
**Hospital stay**, median (IQR)	13 (9–17)	12 (9–17)	13 (9–17)	0.53
**BBS**, median (IQR)	49, (38–53)	49 (40–53)	48 (35–52)	0.33
**TUG** (s), median (IQR)	14,6 (11.4–21.9)	13.5 (10.8–20.0)	16.3 (11.8–23.1)	0.21
**HADS-A**, median (IQR)	4 (1–8)	4 (1–8)	4 (2–8)	0.26

*VESD* Very Early Supported Discharge, *SD* standard deviation, *NIHSS* National Institute of Health Stroke Care, *IQR* interquartile range, *BBS* Berg Balance scale, *TUG* Timed Up and Go, *HADS-A* Hospital Anxiety and Depression scale-Anxiety subscale

^a^ Second day (36–48 h) after arrival at the stroke unit

**Table 2:** Median values for all assessments at the four time points together with the z-values and effect sizes for the changes between the time points

	BBS	TUG (s)	FES(S)	HADS-A
Scores, median (IQR)				
Day 1	50 (38–53)	12.8 (10.2–16.5)	97 (69–103)	3 (0–6)
Month 1	52 (48–55)	11 (9.5–13.8)	122 (92–130)	4 (1–6)
Month 3	53 (47–56)	11.5 (9.2–14.4)	123 (88–130)	2 (0–6)
Month 12	52 (46–55)	11.4 (9.4–15.5)	121 (93–130)	3 (0–6)
Change over time, z-value (effect size)				
Day 1–month 1	−3.445 (0.35)	−3.876 (0.39)	−6.524 (0.67)	− 1.483 (0.15)
Day 1–month 3	−4.040 (0.43)	−4.553 (0.49)	−5.567 (0.58)	− 5.539 (0.59)
Day 1–month 12	−0.253 (0.03)	−3.048 (0.34)	− 1.064 (0.53)	− 0.335 (0.04)
Month 1–month3	−1.489 (0.16)	−2.261 (0.25)	− 0.007 (0.01)	− 5.233 (0.55)
Month 1–month 12	− 2.252 (0.24)	− 0.180 (0.02)	−4.961 (0.12)	−1.321 (0.14)
Month 3–month 12	−3.491 (0.38)	− 2.223 (0.26)	−1.098 (0.12)	− 0.383 (0.04)

*IQR* interquartile range, *BBS* Berg Balance scale, *TUG* Timed Up and Go, *FES(S)* Falls Efficacy Scale Swedish version, *HADS-A* Hospital Anxiety and Depression scale-Anxiety subscale

**Fig. 2:**
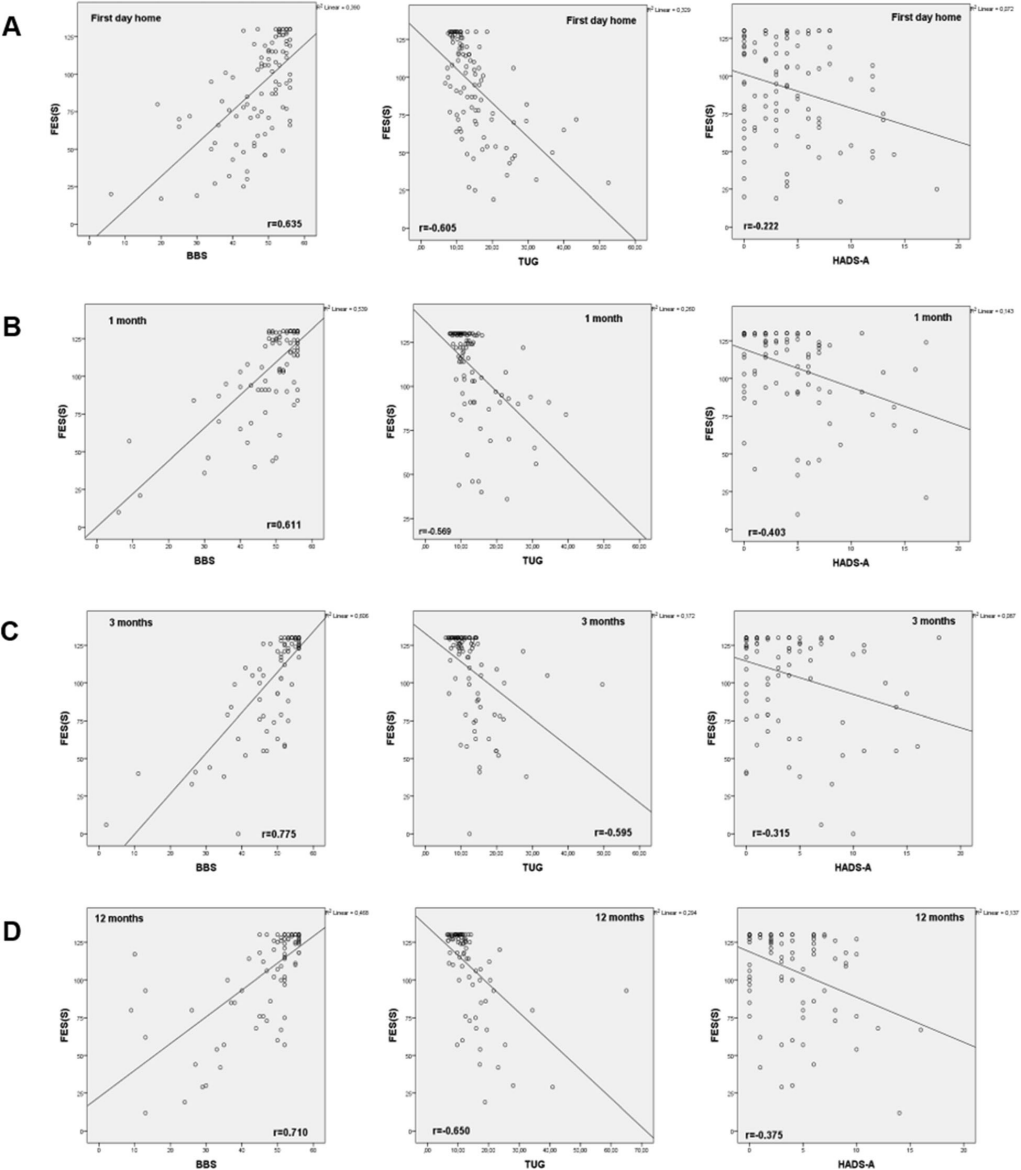
Spearman’s correlation coefficient (r) between the FES(S), BBS, TUG and the HADS-A respectively, at first day home (**a**), 1 month after discharge (**b**), and 3 months (**c**) and 12 months (**d**) post stroke. FES(S) = Fall Efficacy Scale Swedish version; BBS = Berg Balance Scale; TUG = Timed Up and Go; HADS-A = Hospital Anxiety and Depression Scale-Anxiety subscale

The FES(S) showed a large correlation with the BBS and TUG scores on all measurement occasions (Fig. 2, Table 3), with the BBS showing a slightly larger correlation than the TUG test. On the first day after discharge, there was a small correlation (*r* = − 0.222) between anxiety and confidence in postural balance, but at 1 month, 3 months and 1 year post stroke, there was a medium correlation (Fig. 2, Table 3).

**Table 3:** Correlations between the FES(S)/BBS, FES(S)/TUG, and the FES(S)/HADS-A

Assessment	FES(S)/BBS	***p***	FES(S)/TUG	***p***	FES (S)/HADS-A	***p***
**First day home**	0.635	< 0.001	− 0.605	< 0.001	- 0.222	0.03
**1 month after discharge**	0.611	< 0.001	−0.569	< 0.001	- 0.403	< 0.001
**3 months after discharge**	0.775	< 0.001	−0.595	< 0.001	- 0.315	< 0.001
**12 months after discharge**	0.710	< 0.001	−0.650	< 0.001	- 0.375	< 0.001

*FES(S)* Falls Efficacy Scale Swedish version, *BBS* Berg Balance scale, *TUG* Timed Up and Go, *HADS-A* Hospital Anxiety and Depression scale-Anxiety subscale

There was a statistically significant group difference in terms of FES(S) at the 1-month and 3 month follow-ups, with the control group reporting poorer confidence in postural balance ability than the intervention group (Table 4). In the assessment with the BBS, there was a significant difference between the groups at the 3-month follow-up, with the control group having more impaired postural balance than the intervention group (Table 4). In the assessment with the TUG test, there was no significant difference between the groups on any of the measurement occasions (Table 4). In the comparison of the correlation between the BBS and FES(S) scores in the two groups on each occasion, there was no significant difference at any time point (Table 5). Regarding the correlation between the TUG and FES(S) scores in the different groups, there was a significant difference between the VESD group and the control group in favour of the VESD group at the 1-month follow-up (Table 5). There was no significant difference in the correlation between postural balance confidence and anxiety between the two groups, but there was a tendency for a lower correlation in the control group (Table 5). There were no harmful or unintended effects reported in the groups.

**Table 4:** The BBS, TUG, FES(S) and the HADS-A scores at the different follow-ups in the VESD and control groups

Assessment	BBS			TUG (sec)			FES(S)			HADS-A		
Group affiliation	VESD	Control	***p***	VESD	Control	***p***	VESD	Control	***p***	VESD	Control	***p***
**First day home, median (IQR)**	51 (47–55)	49 (43–54)	0.18	11.7 (9.8–13.3)	12.9 (10.9–16.8)	0.42	100 (72–125)	94 (62–121)	0.282	3 (0–5)	4 (1–7)	0.15
**1 month after discharge**	54 (49–56)	51 (46–55)	0.14	10.8 (9.2–13.3)	11.3 (9.9–15.3)	0.17	124 (104–130)	114 (80–129)	0.015	3 (0–6)	5 (1–7)	0.22
**3 months after discharge**	54 (51–56)	51 (45–56)	0.05	10.7 (8.9–14.0)	12.2 (9.6–15.4)	0.15	126 (110–130)	109 (72–130)	0.016	1 (0–5)	4 (1–7)	0.55
**12 months after discharge**	52 (48–55)	51 (45–55)	0.19	10.8 (9.1–15.3)	11.8 (10.0–15.8)	0.49	124 (97–130)	118 (82–130)	0.753	3 (0–6)	3 (1–6)	0.78

*BBS* Berg Balance scale, *TUG* Timed Up and Go, *FES(S)* Falls Efficacy Scale Swedish version, *HADS-A* Hospital Anxiety and Depression scale-Anxiety subscale, *VESD* Very Early Supported Discharge, *IQR* interquartile range

**Table 5:** Correlations between self-estimated postural balance, assessed postural balance and anxiety depending on group affiliation

Time for assessment	FES(S)-BBS, VESD/Control	***p***	FES(S)-TUG VESD/Control	***p***	FES(S)/HADS-A VESD/Control	***p***
**First day home**	0.712^***^/0.594^***^	0.15	- 0.565^**^/−0.655^**^	0.24	- 0.337^*^/− 0.113	0.12
**1 month after discharge**	0.641^***^/0.599^**^	0.37	- 0.709^**^/−0.416^**^	0.02	- 0.358^**^/−0.382^**^	0.45
**3 months post-stroke**	0.723^**^/817^**^	0.14	- 0.621^**^/−0.554^**^	0.32	- 0.437^**^/−0.193	0.11
**12 months post-stroke**	0.741^***^/704^**^	0.46	- 0.584^**^/−0.514^**^	0.32	- 0.444^**^/−0.259	0.17

*FES(S)* Falls Efficacy Scale Swedish version, *BBS* Berg Balance Scale, *TUG* Timed Up and Go, *HADS-A* Hospital Anxiety and Depression scale-Anxiety subscale, *VESD* Very Early Supported Discharge

^***^p < 0.0001, ^**^p < 0.001, ^*^p < 0.05

## Discussion

This study explored the association between confidence in postural balance, observer-assessed postural balance, and anxiety after mild stroke. A second aim was to investigate whether the intervention with VESD affected a possible association between self-confidence in postural balance, observer assessed postural balance and anxiety. To our knowledge, this is the first longitudinal study examining these questions during the first year after stroke. Our results showed that there was a large correlation between postural balance confidence and observer-assessed postural balance, which we interpret to be due to the majority of patients with stroke seemingly having realistic insight into their impairment of postural balance. These findings are in line with previous reports that individuals with poor postural balance after a stroke are at risk for reduced confidence in their postural balance in the first year of community reintegration when compared with that of age-matched individuals without stroke [7]. Self-efficacy is defined as “an individual’s judgement of his or her ability to organize and execute given types of performances” [41], and Bandura’s theory of self-efficacy predicts that the ability to perform tasks depends on both physical ability and mental confidence or self-efficacy [49]. If we interpret our results based on this reasoning, patients with mild stroke can reliably assess their ability to perform activities in daily life. When performing the correlation analysis according to group affiliation, we could not show any difference when postural balance was assessed with BBS, but the analysis between the TUG and FES(S) scores showed a significant difference between the VESD group and the control group in favour of the VESD group at the 1-month follow-up. A possible reason for this could be that the intervention, which lasted for at most 1 month and involved training in various activities in the patients’ home environment, gave the patients better insight into their postural balance. One month after discharge, similar to the control group, the patients in the intervention group no longer had any support from the VESD team and perhaps became less active and therefore less aware and able to estimate their postural balance in daily activities. Since earlier studies have concluded that ESD can reduce long-term dependency and admission to institutional care as well as reduce the length of hospital stay, and we now conclude that there is no difference in the correlation depending on group affiliation; thus, we can only agree with those studies.

In the acute stage, there was only a small correlation between postural balance confidence and anxiety, but after a month and up to 1 year post stroke, there was a moderate correlation. This finding is consistent with the conclusion of Hellström et al. based on their evaluation of the FES(S) to measure clinically meaningful changes over time for patients in the post-acute state [44]. Why the correlation in this study is small in the acute stage is unclear, but perhaps the patients in the acute stroke stage are not aware of their symptoms and the impact of their symptoms on activities in daily life, which previous studies have also found and discussed [50]. It is also possible that in the emergency phase, there is so much else for patients to be anxious about that the feeling of impaired postural balance may be less important as a source of anxiety.

We could not show any significant correlation between the FES(S) and HADS-A scores on any of the assessment occasions. Earlier studies have concluded that the results on a correlation between anxiety and fall efficacy are mixed, and evidence is insufficient to draw a conclusion about this relation [51]. The majority of the studies in this review and similar reviews, however, have only included populations not affected by specific medical characteristics. This makes comparison with our results difficult, even though one can imagine that the same mix of results is found in the stroke population. Further studies regarding this relationship in stroke populations would therefore be of interest.

This study’s sample size was relatively large, and patients were included and assessed in the acute stroke and followed up at four subsequent time points, which is a strength. There was no significant difference between the groups at baseline, which is also a strength.

A limitation is that the data were extracted from a study that included only patients with mild stroke, but the intention was to assess patients with mild-to-moderate stroke. It might be that the increased use of thrombolysis and thrombectomy together with increased primary and secondary prevention has led to fewer massive strokes and more mild-to-moderate strokes [52]. Thus, the implications of our results are limited to this specific group of patients. The patients in the current study had discretely impaired postural balance in the acute stage and no possibility of significant improvement. This was shown by the effect size for the BBS, which varied between 0.03 and 0.43 between all the assessment occasions. The same was true for the TUG test: the effect size varied between 0.02 and 0.49, which shows only a low-medium probability that there was a change in the TUG score. This was also probably due to the overall mild stroke severity in the study participants and consistent with the generally mild stroke suffered by patients in Sweden [53].

A possible bias is that we did not take into account any previous neuropsychological symptoms that were present before the stroke. We have took into account the location of the stroke but could not find any correlations. It has been suggested that there is an association between post-stroke depression (PSD) and left hemisphere damage, but this association has not been adequately or consistently confirmed. It has been noted that other lesion variables were not examined and that it is possible that these variables may also play a role in the development of PSD [54]. One could imagine that this could also play a role in the development of post-stroke anxiety. Other psychiatric post stroke comorbidities, e.g., post-stroke emotional incontinence [55] and post-stroke anger aggressive behaviour [56], that can have influenced the results, were not assessed. Further research in this matter would be of interest.

The median patient age in this study was 74 years, which also limits the implications of our results. The dropout percentage was 24–34%, which is rather large and a limitation of the study. To decrease the number of dropouts, patients who were lost to follow-up due to loss of contact were invited to the next follow-up.

## Conclusion

Patients with mild stroke seem to be able to assess their ability to perform activities in daily life without falling. Examining this ability can provide important and useful information in the planning of rehabilitation and to support the patients to dare to be physically active after discharge from the hospital. The VESD intervention did not affect this ability among patients in this study; on the contrary, the intervention group was slightly better than the control group at estimating their postural balance ability in line with objectively measured postural balance on some occasions.

## Data Availability

According to the Swedish regulations shown in https://etikprovning.se/for-forskare/ansvar/, the complete dataset cannot be made publicly available for ethical and legal reasons. Interested researchers can request access to the data by emailing the authors (contact: ks.sunnerhagen@neuro.gu.se).

## Abbreviations

ADL: Activity of Daily Living
BI: Barthel Index
ESD: Early Supported Discharge
FES(S): Falls Efficacy Scale-Swedish version
FMA: Fugl Meyer Assessment
GOTVED: Gothenburg Very Early Supported Discharge
HADS: Hospital Anxiety and Depression Scale
IQR: Interquartile range
MoCA: Montreal Cognitive Assessment
NIHSS: National Institute of Health Stroke Scale
VESD: Very Early Supported Discharge
WHO: World Health Organization
